# Peptide LCGA-17 Attenuates Behavioral and Neurochemical Deficits in Rodent Models of PTSD and Depression

**DOI:** 10.3390/ph15040462

**Published:** 2022-04-12

**Authors:** Anton V. Malyshev, Iuliia A. Sukhanova, Valeria M. Ushakova, Yana A. Zorkina, Olga V. Abramova, Anna Y. Morozova, Eugene A. Zubkov, Nikita A. Mitkin, Vsevolod V. Pavshintsev, Igor I. Doronin, Vasilina R. Gedzun, Gennady A. Babkin, Sergio A. Sanchez, Miah D. Baker, Colin N. Haile

**Affiliations:** 1 Lactocore Inc., Newton, MA 02460, USA; malyshev@lactocore.com (A.V.M.); mitkin@lactocore.com (N.A.M.); pavshintsev@lactocore.com (V.V.P.); doronin@lactocore.com (I.I.D.); gedzun@lactocore.com (V.R.G.); gbabkin3@mail.ru (G.A.B.); 2 Department of Basic and Applied Neurobiology, V.P. Serbsky Federal Medical Research Centre of Psychiatry and Narcology, 119034 Moscow, Russia; ushakovavm@yandex.ru (V.M.U.); zorkina.ya@serbsky.ru (Y.A.Z.); abramova1128@gmail.com (O.V.A.); hakurate77@gmail.com (A.Y.M.); zubkov@ngs.ru (E.A.Z.); 3 Department of Psychology/TIMES, University of Houston, Houston, TX 77204, USA; sanchez.19sergio@gmail.com (S.A.S.); miah.dbaker@gmail.com (M.D.B.); cnhaile@uh.edu (C.N.H.)

**Keywords:** chronic stress, novel treatment, peptide drug, biogenic amines

## Abstract

We have previously described the LCGA-17 peptide as a novel anxiolytic and antidepressant candidate that acts through the α2δ VGCC (voltage-gated calcium channel) subunit with putative synergism with GABA-A receptors. The current study tested the potential efficacy of acute and chronic intranasal (i.n.) LCGA-17 (0.05 mg/kg and 0.5 mg/kg) in rats on predator odor-induced conditioned place aversion (POCPA), a model of post-traumatic stress disorder (PTSD), and chronic unpredictable stress (CUS) that produce a range of behavioral and physiological changes that parallel symptoms of depression in humans. CUS and LCGA-17 treatment effects were tested in the sucrose preference (SPT) social interaction (SI), female urine sniffing (FUST), novelty-suppressed feeding (NSFT), and forced swim (FST) tests. Analysis of the catecholamines content in brain structures after CUS was carried out using HPLC. The efficacy of i.n. LCGA-17 was also assessed using the Elevated plus-maze (EPM) and FST. Acute LCGA-17 administration showed anxiolytic and antidepressant effects in EPM and FST, similar to diazepam and ketamine, respectively. In the POCPA study, LCGA-17 significantly reduced place aversion, with efficacy greater than doxazosin. After CUS, chronic LCGA-17 administration reversed stress-induced alterations in numerous behavioral tests (SI, FUST, SPT, and FST), producing significant anxiolytic and antidepressant effects. Finally, LCGA-17 restored the norepinephrine levels in the hippocampus following stress. Together, these results support the further development of the LCGA-17 peptide as a rapid-acting anxiolytic and antidepressant.

## 1. Introduction

Post-traumatic stress disorder (PTSD) is a disabling condition that occurs in many individuals following exposure to an extremely traumatic event. The probability of developing PTSD is influenced by a number of factors, some of which render certain groups more susceptible to developing the disorder [1,2,3]. The primary symptoms of PTSD include intrusive thoughts, impaired cognition, avoidance, anxiety, sleep disturbances, and heightened reactivity that results in functional impairment accompanied by abnormal physiological measures that relate to stress responsivity [4]. Individuals with PTSD often present with other psychiatric disorders such as anxiety, mood [5,6], and substance use disorders [7,8]. FDA-approved drugs for PTSD treatment include the selective serotonin reuptake inhibitors (SSRIs) sertraline (Zoloft) and paroxetine (Paxil). The current APA PTSD guidelines recommend three SSRIs, fluoxetine, paroxetine, or sertraline, as well as venlafaxine from the class of serotonin norepinephrine reuptake inhibitors (SNRIs) [9]. However, evidence suggests these medications show minimal efficacy with significant side effects leading to noncompliance [3,10,11]. Antidepressants alone are usually ineffective for treating insomnia and the nightmares associated with PTSD [12]. The potential benefit of an optimum pharmacotherapeutic for comorbid conditions (e.g., anxiety and depression) would be a balance between treatment efficacy and potential side effects. Thus, there is an unmet medical need for novel therapeutics to treat PTSD.

Recent research has shown an increased interest in peptides as pharmacological therapeutics. Advances in structural biology have broadened the allure of peptides beyond the treatment of hormonal deficiencies. Chemically, peptides represent a unique class of compounds between small molecules and proteins, yet are biochemically and therapeutically distinct from both. The structural versatility of peptides, together with their superior safety and tolerability profiles, make them promising drug candidates [13].

LCGA-17 is a milk hydrolysate-derived peptide with amino acid sequence Ac-FQSE (Ac-Phe-Gln-Ser-Glu), identified computationally and validated in vitro as a ligand of the α2δ auxiliary subunit of voltage-gated Ca^2+^ channels (VGCC) with putative synergism at GABA-A receptors [14]. We focused on α2δ VGCC and GABA-A receptors as putative targets, because they are established as mediators of efficacious anxiolytics, antidepressants, and anticonvulsants [15,16,17,18,19,20]. Behavioral characterizations in mice using acute intraperitoneal administration of LCGA-17 demonstrated anxiolytic-like effects in the open field test (OFT), elevated plus-maze (EPM), and marble burying test (MBT), as well as antidepressant-like properties in the forced swim test (FST). In addition, anxiolytic effects were produced at high doses without signs of sedation [14].

Here, we aim to expand the peptide characterizations by assessing the effects of LCGA-17 in a well-characterized animal model of PTSD (predator odor-induced conditioned place aversion, POCPA) and a behavioral model of chronic unpredictable stress (CUS). Exposure to POCPA induces a phenotype similar to PTSD in humans by inducing the disease’s key characteristics, including aversion (avoidance behavior) and enhanced and enduring anxiety [21]. Predator odor exposure also dysregulates the hypothalamic–pituitary–adrenal axis (HPA) and alters stress responses [22]. The CUS model involves prolonged exposure to unpredictable stressors that produce a range of behavioral and physiological changes that parallel the symptoms of depression and other mood disorders in humans [23]. CUS is often used to assess potential antidepressant medications [24,25,26].

We decided to use intranasal (i.n.) delivery for LCGA-17. The intranasal route of administration for short peptides has several advantages, such as rapid systemic drug absorption and the potential to more effectively bypass the blood–brain barrier and access the central nervous system [27,28]. For patients, this method is relatively noninvasive and limits the side effects associated with the peripheral administration of substances [29].

Our aim was to evaluate the potential efficacy of i.n. LCGA-17 after acute and chronic administration in POCPA and CUS. We tested avoidance behavior in the POCPA model. After CUS exposure, we analyzed the sucrose preference (SPT), social interaction (SI), female urine preference (FUST), novelty-suppressed feeding (NSFT), and the forced swim (FST) in rats, as well as serotonin, norepinephrine, and dopamine levels in brain structures.

## 2. Results

### 2.1. Dose-Finding Studies in Naïve Rats after Intranasal Administration

**Figure 1:**
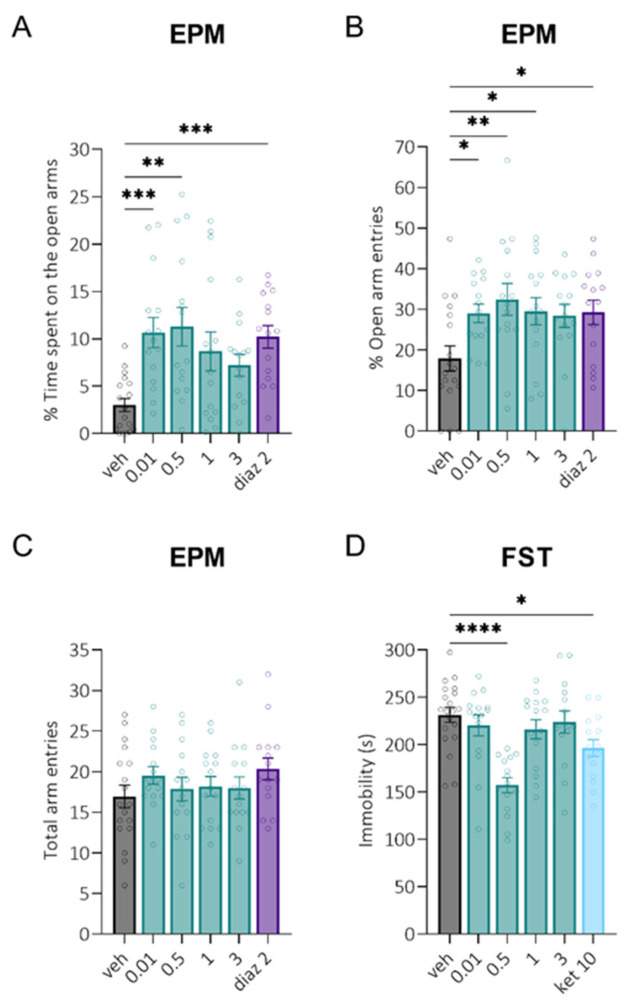
Anxiolytic-like and antidepressant-like efficacy of LCGA-17 after single i.n. administration in rats. Separate groups of animals (*n* = 15–18) were treated with LCGA-17 at the dosages indicated of diazepam (diaz, 2 mg/kg, i.p.) or ketamine (ket, 10 mg/kg, i.p.), followed by behavioral testing 30 min later. (**A**) In the EPM, LCGA-17 increased the percent of time spent on the open arms, with significant effects at 0.5 mg/kg and 0.01 mg/kg (H (5, 87) = 22.6, *p* = 0.0004; K–W, Dunn’s test), similar to diazepam; (**B**) The anxiolytic-like response in the EPM was also reflected as an increased percent of open arms entries, which was significant at 0.5 mg/kg, 0.01, and 1 mg/kg (F (5, 87) = 2.9, *p* = 0.018; ANOVA, Dunnett’s test), similar to diazepam; (**C**) No effects of LCGA-17 or diazepam were observed on total arm entries in the EPM test (F (5, 87) = 0.89, *p* = 0.48; ANOVA); (**D**) In the FST, only in a dose of 0.5 mg/kg LCGA-17 reduced the duration of immobility to a greater extent than ketamine (F (5, 87) = 8.1, *p* < 0.0001; ANOVA, Dunnett’s test). * *p* < 0.05, ** *p* < 0.01, *** *p* < 0.001, and **** *p* < 0.0001 vs. vehicle.

The anxiolytic and antidepressant-like activities of LCGA-17 were tested in the EPM and FST tests. In the EPM test, LCGA-17 and 2 mg/kg diazepam similarly increased the percent of time spent on the open arms of the maze (Figure 1A) and the percent of open arms entries (Figure 1B). LCGA-17-induced anxiolysis was pronounced at 0.5 mg/kg (*p* < 0.01) and 0.01 mg/kg (*p* < 0.05) based on both parameters. At 1 mg/kg, LCGA-17 only increased the percent of open arms entries (*p* = 0.03), and at a dose of 3 mg/kg, the effects of the peptide were absent (*p* > 0.07). Neither LCGA-17 nor diazepam affected the total arm entries (*p* > 0.49), indicating the absence of locomotor effects in this test (Figure 1C). Together, these results suggested that LCGA-17 had rapid anxiolytic-like properties after i.n. administration, which was more prominent at the 0.01–0.5 mg/kg dose range. The peptide effects were similar to diazepam at 0.01–0.5 mg/kg, yet without any sedative effects.

In the FST, 0.5 mg/kg LCGA-17 significantly decreased the immobility time (*p* < 0.0001). This effect was more prominent than the rapid antidepressant ketamine (10 mg/kg, *p* = 0.03) (Figure 1D). However, other doses of LCGA-17 did not significantly decrease the immobility (*p* > 0.68).

Based on the results, two doses of LCGA-17 were tested in the stress models—0.05 mg/kg and 0.5 mg/kg.

### 2.2. POCPA Study

**Figure 2:**
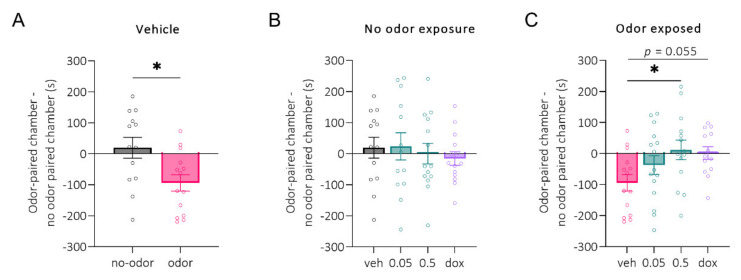
Conditioned place aversion to predator odor in rats treated with LCGA-17 at the dosages indicated or doxazosin (dox, 1 mg/kg). Drugs were administered twice: after conditioning with odor/no odor and the next day 30 min before the test. (**A**) Predator odor induced significant place aversion in the vehicle-treated groups (t_(27)_ = 2.690, *p* = 0.012; two-tailed unpaired *t*-test); (**B**) No odor-conditioned animals showed no place aversion 24 h post-exposure, independent of the treatment (F (3, 50) = 0.29, *p* = 0.83; ANOVA); (**C**) LCGA-17 at a dose of 0.5 mg/kg was more efficient in reducing odor-induced place aversion than dox (F (3, 52) = 2.9, *p* = 0.04; ANOVA, Dunnett’s test). * *p* < 0.05 vs. vehicle odor-exposed group.

Figure 2A shows no odor and odor-exposed groups treated with the vehicle. Predator odor exposure produced a significant conditioned place aversion at 24 h post-exposure (*p* = 0.01). The treatment groups did not differ when the rats were not exposed to a predator odor (Figure 2B). Administration of 0.5 mg/kg (*p* = 0.02), but not 0.05 mg/kg, LCGA-17 significantly attenuated odor-induced place aversion, as observed in the vehicle-treated animals (Figure 2C). Doxazosin (dox, 1 mg/kg) showed efficacy at reducing odor-paired chamber avoidance but only at a trend level (*p* = 0.06, Figure 2C).

### 2.3. CUS Study

#### 2.3.1. SPT2 (Sucrose Preference Test)

**Figure 3:**
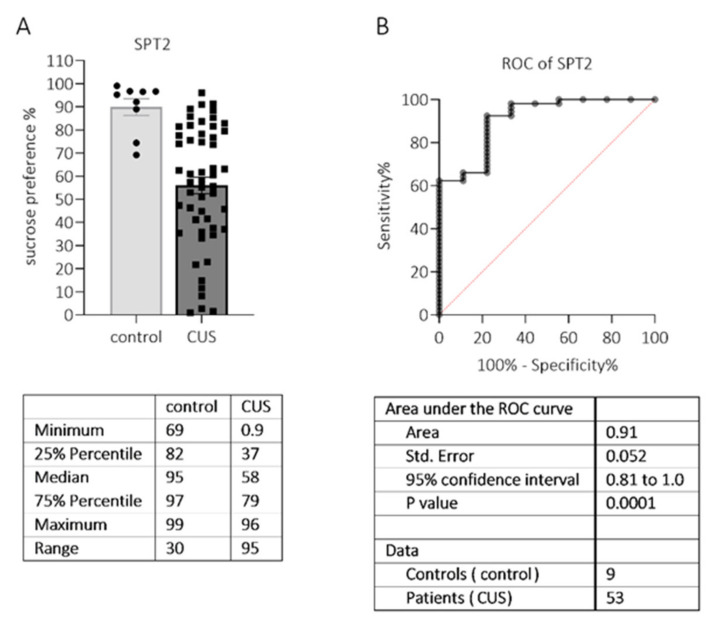
The results of the sucrose preference test (SPT2) after CUS procedures in rats. Control animals (*n* = 9) were living under standard vivarium conditions, while stress-exposed animals (CUS, *n* = 56) were housed individually and exposed to unpredictable stressors (two a day) for 26 days; (**A**) The sucrose preference was significantly reduced in stressed animals (M-W U = 44.0, *p* < 0.0001); (**B**) The ROC curve characteristics showed a high predictive value for a sucrose preference as a marker of stress (AUC value = 0.91).

After 26 days of the CUS procedure, SPT2 was carried out to assess the effects of stress exposure. Interestingly, in the group of stressed rats (*n* = 56), we observed two clusters of animals, one with a high preference for sucrose (≥73.6%, *n* = 20 (38%)) and one with a low preference (≤63.4%, *n* = 33 (62%)) (Figure 3A). The minimal value in the control group was 69% (Figure 3A). In a previous study by Strekalova and colleagues (2004), a threshold of 65% SP was chosen, as none of the control mice had lower preference levels [30]. Four weeks of CUS decreased the SP in 50–70% of mice by ≤65% [31].

We performed a ROC analysis to choose a sucrose preference index cut-off value to divide the rats into stress “vulnerable” and “resilient” (Figure 3B). We chose a cut-off value of <74.2% with a sensitivity = 66.0%, specificity = 88.9%, and likelihood ratio = 5.9. Accordingly, only animals that met the sucrose preference criteria <74.2% were taken for further experiments (*n* = 36, 64.3% of the stressed group).

#### 2.3.2. SI (Social Interaction Test)

**Figure 4:**
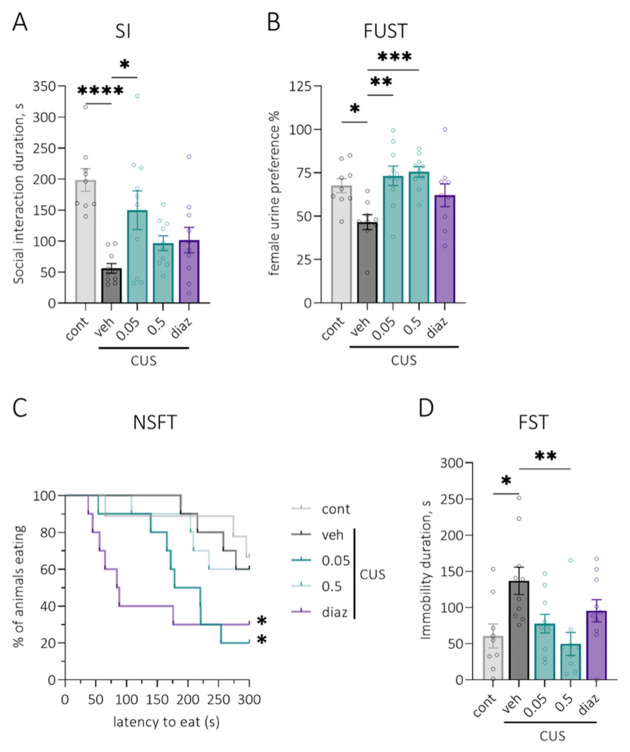
Anxiolytic- and antidepressant-like efficacy of LCGA-17 in the CUS model. Animals were exposed to chronic stress for 26 days, and control rats remained intact. Separate groups of rats (*n* = 9) were treated with i.n. LCGA-17 (0.05 and 0.5 mg/kg) or diazepam (0.5 mg/kg, i.p.) or the vehicle for several days, followed by behavioral analyses. On the day of the experiment, substances were administered 30 min before the test. (**A**) In the SI test, the duration of social contact (s) was significantly decreased in vehicle-treated CUS-exposed rats, while LCGA-17 0.05 mg/kg (4 injections) significantly increased this parameter (H (5, 45) = 19.88, *p* = 0.0005; K–W, Dunn’s test); (**B**) In the FUST, CUS exposure led to a decreased preference for female urine (%); at the same time, LCGA-17 at both doses (8 injections) abolished the effect of stress on reward-seeking behavior (F (4, 40) = 5.62, *p* = 0.001; ANOVA, Dunnett’s test); (**C**) In the NSFT, latency to eat was not affected by stress, though 0.05 mg/kg LCGA-17 and diazepam administered for 11 days both decreased this parameter (chi-square = 11.67, *p* = 0.02; Log-rank Mantel–Cox test); (**D**) In the FST, stressed vehicle-treated rats spent more time immobile (s), while LCGA-17 (18 injections) dose-dependently decreased the immobility in this test (H (5, 45) = 14.99, *p* = 0.005; K–W, Dunn’s test). * *p* < 0.05, ** *p* < 0.01, *** *p* < 0.001, and **** *p* < 0.0001 vs. veh group.

Rats exposed to chronic stress showed fewer social contacts than control animals (Figure 4A) (*p* < 0.0001). Four injections of LCGA-17 at a dose of 0.05 mg/kg significantly increased the durations of social interactions, suggesting an anxiolytic-like effect of the peptide (Figure 4A, *p* = 0.05). At 0.5 mg/kg, LCGA and 0.5 mg/kg diazepam showed no significant changes for the duration of social interactions.

#### 2.3.3. FUST (Female Urine Sniffing Test)

Chronic stress led to a significant decrease (*p* = 0.01) in preference for female urine in male rats, suggesting impaired reward-seeking behavior (Figure 4B). Preference for female urine was restored following eight days of 0.05 mg/kg and 0.5 mg/kg LCGA-17 but not 0.5 mg/kg diazepam administration (Figure 4B, *p* = 0.001 and *p* = 0.0008, respectively). A reduction of hedonic deficit might propose an antidepressant-like activity of the peptide.

#### 2.3.4. NSFT (Novelty-Suppressed Feeding Test)

The latency to eat was not affected by chronic stress. Both 0.05 mg/kg LCGA-17 and 0.5 mg/kg diazepam administered for eleven days significantly reduced hyponeophagia (Figure 4C, *p* = 0.02 and *p* = 0.05, respectively). The results suggest stress-relieving properties of LCGA-17 similar to those of diazepam.

#### 2.3.5. SPT3 (Sucrose Preference Test)

**Figure 5:**
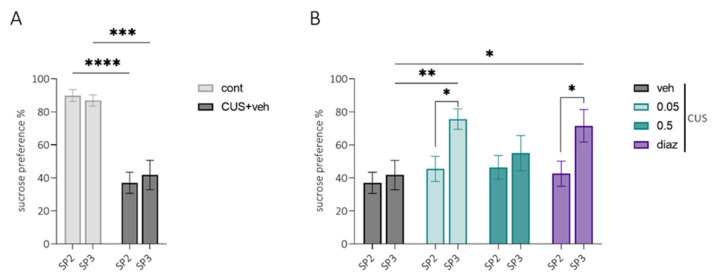
Sucrose preference index (%) of rats several (1–5) days after (SP2) and 3.5 weeks after CUS exposure (SP3). At the SP3 point, animals received 16 injections of either LCGA-17 at a dose of 0.05 and 0.5 mg/kg or diazepam 0.5 mg/kg or the vehicle. (**A**) the sucrose preference index (%) of rats from the control and stressed groups showed treatment effects (F (4, 40) = 11.92, *p* < 0.0001), test effects (F (1, 40) = 10,52, *p* = 0.0025), and treatment × test interactions (F (4, 40) = 2666, *p* = 0.047). CUS exposure resulted in the persistent reduction of sucrose preference in comparison with control groups several days after (SP2, *p* < 0.0001) and 3.5 weeks after stress (SP3, *p* = 0.0002); (**B**) Sucrose preference index (%) of stressed groups of rats that showed a treatment effect (F (4, 40) = 11.92, *p* < 0.0001), test effect (F (1, 40) = 10,52, *p* = 0.0025), and treatment × test interaction (F (4, 40) = 2666, *p* = 0.047). LCGA-17 in a dose of 0.05 mg/kg improved the hedonic behavior, and the same effect was observed in the diazepam-treated group. * *p* < 0.05, ** *p* < 0.01, *** *p* < 0.001, and **** *p* < 0.0001; two-way ANOVA, Sidak’s test.

The high level of anhedonia in stressed animals persisted up to 23 days after SPT2 (Figure 5A). At the onset of SPT3, animals received treatment for 16 days. Improved sucrose preference was observed in groups receiving LCGA-17 at a dose of 0.05 mg/kg or 0.5 mg/kg diazepam compared to the results of SP2 (Figure 5B, *p* = 0.02 and *p* = 0.01, respectively) and compared to the SP3 results in vehicle-treated animals (Figure 5B, *p* = 0.006 and *p* = 0.02, respectively). This result suggests that chronic LCGA-17 may reduce anhedonia-like behavior in animals.

#### 2.3.6. FST (Forced Swim Test)

Prolonged immobility in animals subjected to chronic stress suggests increased behavioral despair. LCGA-17 administered for 18 days dose-dependently reduced the immobility duration in rats, with a significant effect at 0.5 mg/kg (*p* = 0.006). Diazepam (0.5 mg/kg) failed to affect behavioral despair in animals.

### 2.4. Biogenic Amines Concentrations after CUS

**Figure 6:**
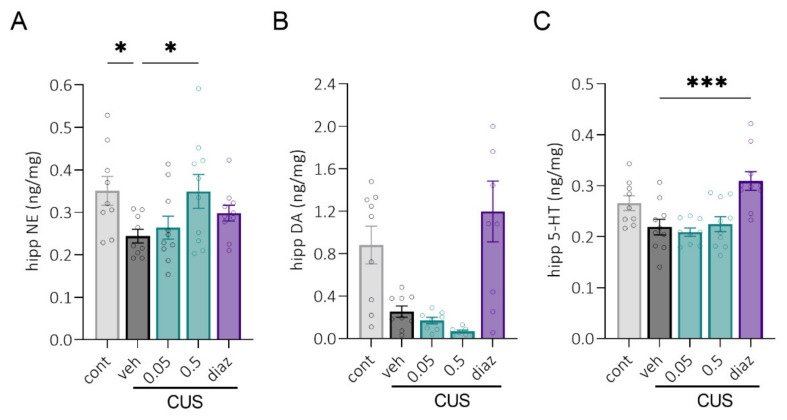
Chronic LCGA-17 effects on the bioamines levels after CUS. Separate groups were treated for 22 days before sacrifice with either LCGA-17 (0.05 and 0.5 mg/kg i.n.) or diazepam (0.5 mg/kg i.p.). (**A**) The norepinephrine level was significantly reduced in the hipp of stressed rats compared to the control animals. LCGA-17 in a dose of 0.5 mg/kg increased the NE levels in the hippocampus to the control levels (F (4, 40) = 2.79, *p* = 0.038; ANOVA, Dunnett’s test); (**B**) None of the treatments showed a significant change in hipp DA concentrations (H (5, 42) = 21.97, *p* = 0.0002; K–W, Dunn’s test); (**C**) Diaz increased the 5-HT levels in the hipp of stressed rats (F (4, 40) = 8.1, *p* < 0.0001; ANOVA, Dunnett’s test). * *p* < 0.05 and *** *p* < 0.001 vs. the veh group.

More than four weeks after CUS, no stress-induced changes in the content of bioamines were found in the prefrontal cortex (PFC) or hypothalamus (Hyp). In the hippocampus (Hipp), there was a decreased level of norepinephrine (NE) in the stressed group compared to the unstressed control group of animals (Figure 6A, *p* = 0.04), whereas the dopamine (DA) and serotonin (5-HT) contents were lower only on a trend level (*p* = 0.1). Chronic LCGA-17 treatment for 22 days at a dose of 0.5 mg/kg restored the NE levels to the control values (Figure 6A, *p* = 0.04). Chronic administration of 0.5 mg/kg diazepam restored the 5-HT levels in Hipp (Figure 6C, *p* = 0.0003) but failed to reach statistical significance for the DA content (Figure 6B, *p* = 0.11).

## 3. Discussion

An estimated 8.4% of adults in the United States had at least one major depressive episode in 2020, and an estimated 3.6% of adults had PTSD [32]. Despite progress in understanding the pathophysiology of stress-related disorders, the development of novel therapeutics has been slow. Classical antidepressant treatments have limited efficacy and substantial safety and tolerability issues. There is an unmet clinical need for the development of rapid-acting antidepressants and anxiolytics.

In the current study, we found that LCGA-17 administered twice i.n. reversed predator odor-induced place aversion, a behavior that replicates stress-induced avoidance behavior in patients with PTSD. We also assessed the potential efficacy of chronic LCGA-17 in the CUS model of depression. Our results indicate that the peptide significantly affected anxiety, depression, and anhedonia in stressed animals. Lastly, we found that LCGA-17 restored the NE levels in the hippocampus after CUS, which may partly underlie its anxiolytic and antidepressant effects. According to the results of the study, LCGA-17 is a promising novel drug candidate for treating psychiatric disorders, including depression and PTSD.

Similarly to what we have previously showed for i.p. administration [14], acute i.n. administration also produced rapid anxiolytic and antidepressant effects in EPM and FST comparable to 2 mg/kg diazepam and 10 mg/kg ketamine, respectively. The anxiolytic response in the EPM was evaluated as an increased percentage of open arms entries and increased time spent on the open arms. As described previously, both parameters were affected by the comparison drug diazepam [33]. The antidepressant response in the FST was evident by decreased immobility duration. The same effect was shown after acute ketamine administration in both rats and mice [34,35,36,37]. We found the most effective anxiolytic response with 0.01 mg/kg and 0.5 mg/kg LCGA-17 and less pronounced at 1 mg/kg, with no effects at 3 mg/kg. Based on the results, we further assessed the two doses of LCGA-17 in two well-characterized stress models.

We tested the efficacy of LCGA-17 as an antidepressant and anxiolytic in an animal model of PTSD—POCPA. Predator odor exposure produced a significant place aversion that was attenuated by LCGA-17. Importantly, LCGA-17 was administered twice, immediately after odor presentation and the next day before the test. We consider this effect of LCGA-17 as anxiolytic-like. Furthermore, the rapid effects of LCGA-17 were greater than those of doxazosin, a competitive alpha1 antagonist that was previously shown to alleviate PTSD symptoms in humans [38,39]. The current results propose that acute LCGA-17 administration after stress exposure had stress-relieving properties at a dose of 0.5 mg/kg with an efficacy similar to or greater than the clinically used drug doxazosin. The scheme of LCGA-17 administration suggests that the tetrapeptide may also affect fear consolidation. Both the consolidation and extinction of conditioned fear are dependent on L-type VGCCs producing long-term potentiation (LTP) in the amygdala. Impaired Ca_v_1.3 functioning leads to an inability to consolidate contextually conditioned fear in mice [40], and the blockade of L-VGCCs in the lateral amygdala impairs the acquisition of long-term auditory conditioned fear [41]. Thus, LCGA-17 may exert its stress-relieving functions as an α2δ VGCCs inhibitor by affecting fear memory processing.

CUS exposure for 26 days resulted in persistent depressive-like behavior. According to the SPT, we observed anhedonia as measured by a decrease in the sucrose preference that endured for more than three weeks after discontinuing the stress. Anxiety-like and depressive-like behaviors of stressed animals were also evident in the SI, FUST, and FST. These results are consistent with previous studies of the effects of chronic stress exposure in various animal models of depression [42,43]. We did not observe an increased latency to eat in the novelty-suppressed feeding test, even though this test is sensitive to chronic stress [44]. Our findings show that chronic LCGA-17 administration ameliorated anxiety- and depressive-related behaviors following CUS in rats in all behavioral paradigms utilized. The peptide increased social interaction in the SI test, normalized sucrose intake in the SPT, produced anxiolysis in the novel environment in the NSFT, and reduced behavioral despair in the FST. In stressed animals, similar effects were observed after chronic clomipramine [42] and fluoxetine [45] treatment. The comparison drug diazepam (0.5 mg/kg), administered chronically, was potent in reducing anxiety in the NSFT. This effect was previously described for mice after CUS [46]. We also observed an enhanced sucrose preference in SPT after diazepam treatment. This effect was previously shown for treatments with chronic diazepam and fluoxetine [47] and acute and chronic ketamine [48]. Thus, the behavioral effects of LCGA-17 were greater than that of diazepam. Benzodiazepines (BZD) can be prescribed to patients with depression in combination with antidepressants or as a monotherapy. Even though BZD may elevate mood, they exert limited effects on the core symptoms of depression [49]. Our results have shown that, unlike diazepam, LCGA-17 showed a potent efficacy in alleviating anxiety-like and depressive-like behaviors in rats after chronic stress.

Chronic stress resulted in a long-term decline of bioamines in the hipp, but not the PFC and hyp, more than four weeks after stress exposure. In our study, chronic diazepam was potent at restoring the 5-HT levels in the hippocampus of stressed rats. Our results are comparable to a previous study showing that nineteen-day repeated administration of diazepam increased hippocampal 5-HT and striatal DA, which may be responsible for the antianxiety effect of the drug [50].

The hipp is part of limbic system circuitry that affects mood and memory formation. Dysregulation of hippocampal functions accompanies such mental disorders as depression, PTSD, and anxiety [51]. After chronic stress in animals, behavioral impairments are associated with prolonged activation of the HPA axis, resulting in elevated glucocorticoids and neurotoxic damage to the hippocampus [23]. Previous findings indicate that α2δ ligand gabapentinoids prevent chronic stress-induced depression-like behavior and promote hippocampal neurogenesis [52]. Thus, the pharmacological modulation of hippocampal functioning might be beneficial for treating stress-induced disorders.

LCGA-17 restored the NE levels in the hipp of stressed animals. In a recent study, exposure to single prolonged stress decreased the NE levels in the hipp [53]. Therefore, restoring NE concentrations in the hipp may play a role in LCGA-17’s ability to attenuate abnormal behaviors following chronic stress. In addition, the increase of the neurotransmitter concentration in the synaptic cleft may activate signaling pathways via the noradrenergic system, mediating cell proliferation and synaptic plasticity [54]. Indeed, studies have shown that the α2δ ligand gabapentin increases locus coeruleus (LC) neuronal activity and NE release [55]. LC projections go to many parts of the brain, including the hipp. In addition, recent evidence suggests that the gabapentin anxiolytic effect involves rapidly increased the tonic inhibition of neurons by δ subunit-containing GABA-A receptors (Yu et al., 2019). Thus, the same neural activity may relate to potential mechanisms of action of LCGA-17 as well.

The study has the following limitations. (1) The study was carried out in male rats. In humans, mood disorders are more prevalent and typically more severe in women. This has also been reported in preclinical rodent models, in which female animals show great-er effects. (2) Intranasal administration of the peptide has several advantages, which were indicated in the Introduction. However, a dosage could be inaccurate due to the mechanical loss of the injected drug. (3) Animal models of neurological diseases have limited translational potential to human conditions, though preclinical studies in animals are an important tool in biomedical research. (4) The experiments were designed to reduce the number of animals. Same rats were used in the battery of the behavioral test, and the treatment days differed from test to test.

## 4. Materials and Methods

### 4.1. Animals and Housing

Animal experiments were conducted in accordance with the 2011 NIH Guide for the Care and Use of Laboratory Animals. Initially, 95 male Sprague–Dawley rats (Charles River, Wilmington, MA, USA) were used for dose-finding studies. In this series, the behavior of the animals was tested in the EPM and FST. An additional 120 male rats were utilized for the POCPA study. All animal procedures were approved by the University of Houston Institutional Animal Care and Use Committee. For the CUS study, a total of 110 male Wistar rats (“Stolbovaya” laboratory animal nursery, Russia) were used, and the LLC “Lactocore” Ethical Committee approved all procedures. In this series, the behaviors of the animals were tested in a battery of behavioral tests (SPT, SI, FUST, NSFT, and FST). All animals from the experimental groups were tested in each procedure, with at least two days between each test.

Rats were initially housed 3–5 in polypropylene cages within a temperature- and humidity-controlled vivarium maintained on a 12:12 light/dark cycle. Rats weighed about 250–350 g at the start of the experiment and were at least 100 days old. Food and water were available ad libitum throughout the studies.

### 4.2. Drug Treatment

Control groups of the animals received the vehicle (sterile saline) i.n. according to the scheme of administration of the tested drugs in each series.

#### 4.2.1. Dose-Finding Study

Diazepam (diaz, Sigma-Aldrich, St. Louis, MO, USA, 2 mg/kg) and ketamine (Miller Veterinary Supply, Fort Worth, TX, USA, 10 mg/kg) were prepared on the day of the experiment and administered intraperitoneally (i.p.). Four doses of LCGA-17 (0.01, 0.5, 1, and 3 mg/kg, CS, Menlo Park, CA, USA) were prepared fresh each day in sterile saline and administered i.n. 30 min prior to testing. Each dose was administered in a randomized order across rats. Before dosing, the rats were lightly anesthetized (until the loss of the righting reflex) with isoflurane (2.5%, 0.5 L/min oxygen) and placed in the recumbent position. A thin plastic pipette (Fisher Scientific, MA, USA) was inserted into the nostril, and the peptide was administered based on weight (mg/kg) for a total volume of 20 µL, 10 µL per nostril.

#### 4.2.2. POCPA Study

Doxazosin (dox, Sigma-Aldrich, St. Louis, MO, USA, 1 mg/kg, i.p.) and two doses of LCGA-17 (0.05 and 0.5 mg/kg, i.n., CS, Menlo Park, CA, USA) were prepared fresh for each test day in sterile saline and administered i.n.

#### 4.2.3. CUS Study

Diazepam (diaz, Relanium^®^, Polfa, Warsaw, Poland 0.5 mg/kg) was prepared daily and administered i.p. LCGA-17 (0.05 and 0.5 mg/kg, CS, Menlo Park, CA, USA) was prepared fresh each day in sterile saline and administered i.n. 30 min prior to testing. When there were no tests, the substances were administered in the morning. The number of administrations of the studied substances at the beginning of behavioral testing is listed in Table 1.

### 4.3. Behavioral Testing

The EPM was used to assess anxiolytic-like drug activity. For the EPM [56], the percentage of time spent in the open arms, the percentage of open arm entries, and the number of total arm entries during a 5 min session were recorded. The FST assessed antidepressant-like drug activity, as described by Reference [57]. Briefly, on day one, the rats were exposed to a 15 min pre-swim in a cylinder filled with 25 ± 1 °C water. They were re-exposed to the same apparatus for a 5 min test session, with the behavior video recorded the next day. Animal behavior for the EPM and FST were recorded and analyzed with specialized software (Ethovision XT12, VA, USA). The SPT was performed as described previously [58]. Briefly, rats were given for 48 h a free choice between two bottles, one with a 1% sucrose solution and another with tap water. The SPT was carried out three times: first (SPT1), at the baseline prior to any manipulations; second (SPT2), to assess the effect of CUS exposure; and third (SPT3), as part of the behavioral battery of tests. Sucrose consumption in animals was assumed as a measure of anhedonia, a core symptom of major depression according to DSM-5 [59]. The preference index was calculated using the formula:

$$\frac{Volume\;of\;sweet\;water\;consumed}{Total\;volume\;of\;liquid\;consumed}\times 100\%$$

The SI test was performed as previously described [58,60]. Briefly, a juvenile male rat was placed into a home cage of a tested rat for 10 min. The time of social interactions between experimental animals (following, grooming, and sniffing) was recorded. The social interaction test is sensitive to the anxiolytic properties of GABAergic agonists, such as benzodiazepines, ethanol, and barbiturates. Chronic administration of antidepressants have been shown to be ineffective in this test [61]. The NSFT was carried out as previously described [62]. Briefly, a single pellet of regular chow was placed in the center of the open field apparatus. After food deprivation for 24 h, the rats were individually placed in the arena. The time before the onset of eating the food pellet was recorded up to 5 min as the feeding latency in the novel environment. Animals showing a high level of anxiety do not usually approach the treat. Anxiolytic drugs significantly reduce the time to start eating [63]. The FUST was used to evaluate anhedonia, sexual motivation, and exploratory behavior associated with a depressive-like conditions in rodents [64]. Briefly, rats were subjected to the following procedure: (1) 3 min exposure to the cotton tip dipped in water, (2) a 45 min interval, and (3) 3 min exposure to the cotton tip dipped in fresh urine collected from female rat in the estrus phase. The duration of female urine sniffing time was scored. To determine the preference, an index was calculated using the formula:

$$\frac{Time\;spent\;sniffing\;urine}{Total\;sniffing\;time}\times 100\%$$

All experimenters were blind to the treatment groups. Behavior was video recorded and later analyzed with Ethovision XT12.

### 4.4. Predator Odor Conditioned Place Aversion (POCPA) Model

**Figure 7:**
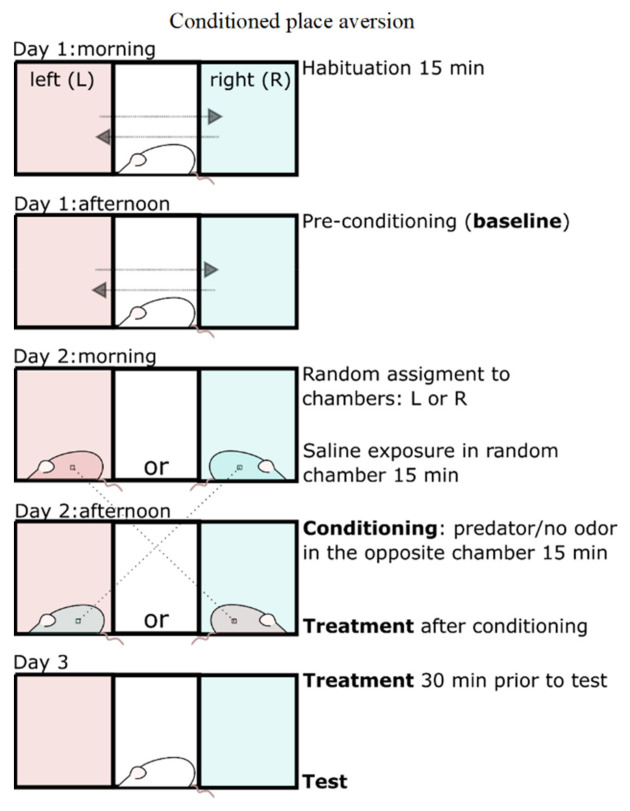
Scheme of the POCPA experiment.

The POCPA protocol was based on previously published studies [22,65,66]. The scheme of the experiment is shown in Figure 7.

Rats were exposed to no odor (sterile saline) or predator odor (bobcat urine, PMart, Sandy Point, ME, USA) using a place conditioning apparatus (MED Associates, Fairfax, VT, USA) consisting of two compartments (20 × 20 × 28 cm) that differed in both visual (wall color) and tactile (floor texture) cues connected by a smaller middle compartment (13 × 20 × 28 cm). Compartments were divided by automated guillotine doors. A nonbiased conditioning method was used so that rats did not prefer one chamber over another. Between 8 and 10 a.m., rats were placed in the middle compartment. The guillotine doors were raised, and the rats were allowed to explore the conditioning apparatus for 15 min (habituation). The time in each compartment and the amount of activity was recorded via infrared sensors and tabulated with commercially available software (MED Test, ver 4.2.0.0, MED Associates, Fairfax, VT, USA). Between 2 and 4 p.m., rats were again allowed to explore the conditioning apparatus, and time and activity were recorded (preconditioning baseline). Twenty-four hours later, rats were randomly assigned to a chamber and exposed to saline while confined to the conditioning compartment for 15 min. In the evening of the same day, rats were confined in the opposite compartment and exposed to predator odor (or no odor) for 15 min. During the pairings, a 5 × 5 cm piece of filter paper was infused with 5 mL of saline or bobcat urine and put under the grid floor of the chamber. The day after predator odor stress, rats were placed in the center compartment and allowed to explore the entire apparatus for 15 min. Rats did not have direct access to the bobcat urine. The entire apparatus was cleaned with disinfectant after each conditioning session. Assessed parameters were zone time (in seconds), preconditioning baseline, and zone time test (in seconds). The place aversion parameters (time difference between baseline and test session) were calculated as follows:

$$Time\;\left[odor\;or\;no\;odor\;paired\;chamber\right]-Time\;\left[baseline\right];$$

where >0 indicates place preference and <0 place aversion.

### 4.5. Chronic Unpredictable Stress (CUS) Model

CUS model was adapted from Willner et al. [26]. The experimental design is shown in Table 1. Rats were group-housed (3–5 animals per cage) for two weeks prior to the experiment. At the onset of the experiment, the animals were placed in individual cages to perform a sucrose preference test (SPT1). Animals with an SP1 index lower than 65% were excluded from the experiment [30]. Animals that met the selected criteria were divided into a group of intact controls (*n* = 10) and a group subjected to chronic unpredictable stress (CUS) (*n* = 53) for 26 days. The stressors are listed in Table 2. After CUS exposure, the animals were retested for sucrose preference (SPT2). Thirty-six rats with sucrose preference (SP) < 74.2% were selected for further experiments. Animals for treatment were divided into groups of nine so that the mean SP was equal in each treatment group. One rat was excluded from the native control group (*n* = 9) due to low sucrose preference values (<65%).

### 4.6. Biogenic Amines Assay

#### 4.6.1. Brain Samples Preparation

Brain samples were obtained after euthanasia and decapitation on days 79 and 80. Samples of the prefrontal cortex, hippocampus, and hypothalamus were dissected from the brain; snap-frozen in liquid nitrogen; and stored at −80 °C before sample preparation. In total, samples from 45 animals were obtained (*n* = 9 in each treatment group). The biogenic amine content was analyzed using high-performance liquid chromatography (HPLC) analysis.

#### 4.6.2. HPLC Analysis

HPLC measurements of brain structures NE, DA, and 5-HT were carried out as described [67].

Briefly, the samples were weighed on an analytical scale as fast as possible to avoid defrosting. Then, 0.1 M HClO_4_ with internal standard 3,4-dihydroxybenzylamine hydrobromide (DHBA, Sigma-Aldrich, MA, USA) was added to the tissue tube. Then, the samples were homogenized using an ultrasonic homogenizer and centrifuged for 10 min at 10,000 rpm, +4 °C. Next, the supernatant was collected and transferred to centrifugal filters (Durapore, Millipore, MA, USA0.22 μm) and recentrifuged for 1 min at 10,000 rpm, +4 °C. For analysis, 10 μL of the obtained sample was used.

In addition to the internal standard (DHBA), external standards were also used in the analysis: solutions of norepinephrine, dopamine, and serotonin (NE, DA, and 5-HT, Sigma-Aldrich, MA, USA) at known concentrations. External and internal standards, each 100 ng/mL, were prepared fresh each day by serial dilutions in 0.1 M HClO_4_.

**Figure 8:**
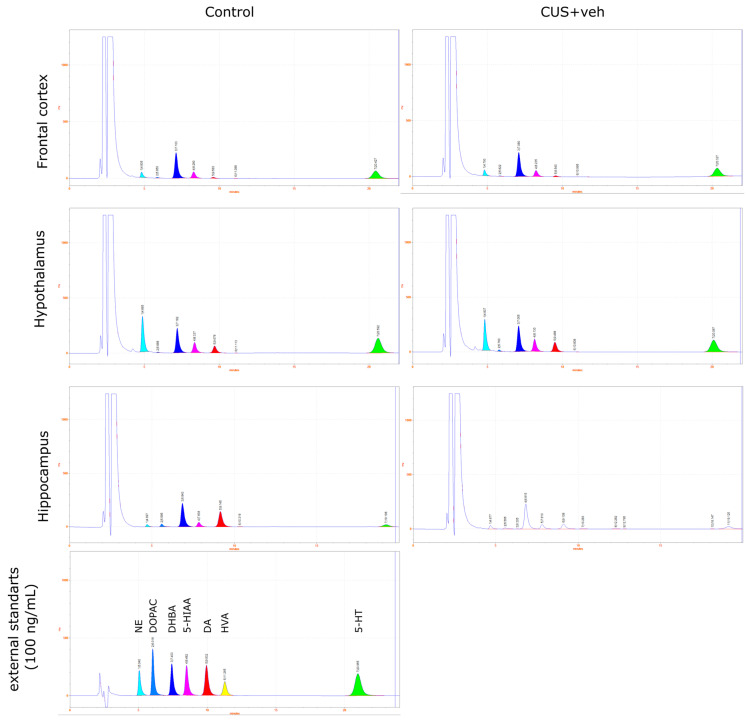
Example chromatograms of monamines NE, DA, and 5-HT and their metabolites (DOPAC, 5-HIAA, and HVA) extracted from the frontal cortex, hypothalamus, and hippocampus after CUS exposure and vehicle treatment.

**Figure 9:**
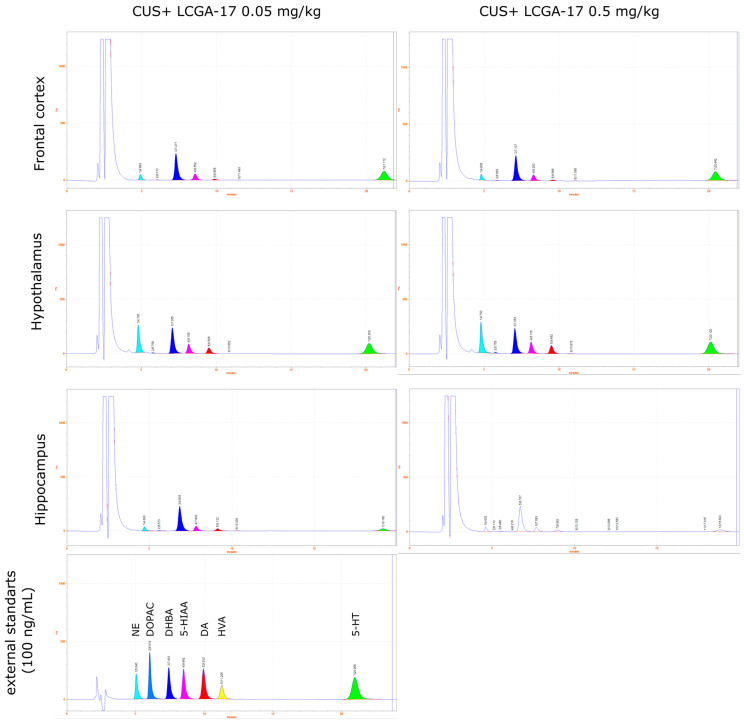
Example chromatograms of monamines NE, DA, and 5-HT and their metabolites (DOPAC, 5-HIAA, and HVA) extracted from the frontal cortex, hypothalamus, and hippocampus after CUS exposure and treatment with LCGA-17 (0.05 mg/kg and 0.5 mg/kg).

**Figure 10:**
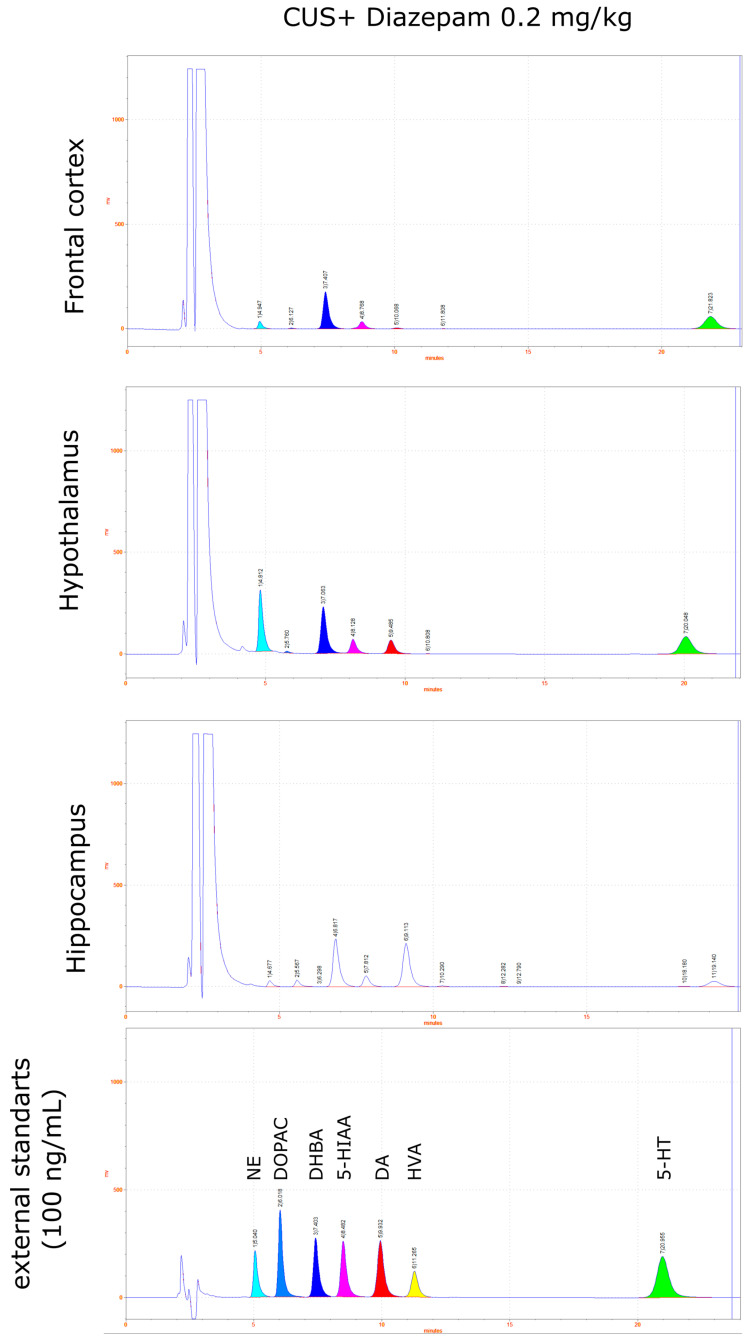
Example chromatograms of monamines NE, DA, and 5-HT and their metabolites (DOPAC, 5-HIAA, and HVA) extracted from the frontal cortex, hypothalamus, and hippocampus after CUS exposure and treatment with diazepam (0.2 mg/kg).

Measurements of monamines in the tissue samples were performed using HPLC with electrochemical detection (HTEC-500, Eicom, Kyoto, Japan) with a carbon electrode WE-3G (Eicom, Kyoto, Japan) using +650 mV applied potential. The system was equipped with a reverse-phase column C-18 SA-50DS (Eicom, Kyoto, Japan) at a flow rate of the mobile phase 200 μL/min. The main electrode was carbon WE-3G (Eicom, Kyoto, Japan), and the reference electrode was Ag/AgCl. The composition of the mobile phase was 0.1 M phosphate buffer, 0.17 mM EDTA, 1.8 mM sodium octosulfonate, and 18% (*vol*/*vol*) methanol, pH 4.5. All peaks obtained were normalized to the internal standard DHBA. Concentrations were calculated from the calibration curve of the external standards (1–250 ng/mL) and expressed as ng/mg tissue. Example chromatograms are presented in Figure 8, Figure 9 and Figure 10.

### 4.7. Statistical Analysis

Statistical analysis was performed using Prism 9.1 (GraphPad, CA, USA). Normality of distributions was assessed using the Shapiro–Wilk test. Normally distributed data were analyzed by one-way ANOVA and Dunnett’s post hoc test or two-way ANOVA with Sidak’s post hoc test. Non-normally distributed data were analyzed by Kruskal–Wallis (K–W), followed by Dunn’s post hoc test. The latency to start eating was assessed using Kaplan–Meyer multiplier estimates, considering the censored variables. In this case, to compare groups, we used chi-square criterion with a further Mantel–Cox test. The data were presented as the mean ± standard error of the mean (SEM). Differences between groups were considered significant at *p* < 0.05.

## 5. Conclusions

In the current study, we found that novel tetrapeptide LCGA-17, with a previously shown affinity for the α2δ subunit of VGCCs, exerts significant anxiolytic- and antidepressant-like effects in animal models of PTSD and depression. LCGA-17 was effective after acute and chronic i.n. administration. The rapid onset of anxiolytic-like action and potent antidepressant-like effects of tetrapeptide during chronic administration support the further development of LCGA-17 as a potential treatment for PTSD, anxiety, and depressive disorders.

## Figures and Tables

**Table 1:** Schedule of experimental manipulations and tests.

Procedures	Day of Experiment	Days of Treatment
Adaptation	1–14	-
SPT1	15–21	-
The division into “stressed” and “unstressed” groups		
CUS	22–48	-
SPT2	49–55	-
The division into treatment groups		
SI	61–62	4
FUST	65–66	8
NSFT	67–69	11
SPT3	72–75	16
FST	75–76	18
sacrifice	79–80	22

SPT—Sucrose Preference Test, CUS—Chronic Unpredictable Stress, SI—Social Interaction (Social Interaction test), FUST—Female Urine Sniffing Test, NSFT—Novelty Suppressed Feeding Test, and FST—Forced Swim Test.

**Table 2:** CUS schedule.

Day of Stress	10 a.m.–6 p.m.	6 p.m.–10 a.m.
1, 8, 15, 22	Cold room (1 h)	Intermittent lightning (on/off every 2 h)
2, 9, 16, 23	Cage tilt (45°)	Water deprivation
3, 10, 17, 24	Stroboscopic light	Light overnight
4, 11, 18, 25	Wet bedding	Mouse cage
5, 12, 19, 26	No bedding	Intermittent lighting (on/off every 2 h)
6, 13, 20	6-month-old new rat	Light overnight
7, 14, 21	White noise	Stroboscopic light

## Data Availability

Data is contained within the article.
